# Improving the thermal structure predictions in the Yellow Sea by conducting targeted observations in the CNOP-identified sensitive areas

**DOI:** 10.1038/s41598-021-98994-7

**Published:** 2021-09-30

**Authors:** Kun Liu, Wuhong Guo, Lianglong Da, Jingyi Liu, Huiqin Hu, Baolong Cui

**Affiliations:** 1 Qingdao National Laboratory for Marine Science and Technology, Qingdao, China; 2 Navy Submarine Academy, Qingdao, China

**Keywords:** Physical oceanography, Environmental impact

## Abstract

Targeted observation is an appealing procedure for improving model predictions. However, studies on oceanic targeted observations have been largely based on modeling efforts, and there is a need for field validating operations. Here, we report the results of a field targeted observation that is designed based on the sensitive areas identified by the Conditional Nonlinear Optimal Perturbation approach to improve the 7th day thermal structure prediction in the Yellow Sea. By introducing the technique of cycle data assimilation and the new concept of time-varying sensitive areas, an observing strategy is designed and validated by a set of Observing System Simulation Experiments. Then, the impact of targeted observations was investigated by a choreographed field campaign in the summer of 2019. The results of the in-field Observing System Experiments show that, compared to conventional local data assimilation, conducting targeted observations in the sensitive areas can yield more benefit at the verification time. Furthermore, dynamic analysis demonstrates that the refinement of vertical thermal structures is mainly caused by the changes in the upstream horizontal temperature advection driven by the Yellow Sea Cold Water Mass circulation. This study highlights the effectiveness of targeted observations on reducing the forecast uncertainty in the ocean.

## Introduction

The predictability of oceanic processes is limited since the ocean is an extremely chaotic dynamic system^1^, the uncertainty of ocean forecasting can be reduced through assimilating observation data^2^. Unlike observations on land, field-deployed oceanic observations are scarce and expensive. Thus, maximizings the individual impact of these limited measurements is a meaningful pursuit. Targeted observation is believed to be a suitable strategy for solving this problem^3–6^.

Interest in the field of oceanic targeted observation has accelerated over the past dozen years, and the effectiveness of oceanic targeted observation has been confirmed by a number of studies^7–13^. However, most of the relevant studies have been largely based on modeling efforts, and experiments in the field are necessary regarding both method validation and the cost-effectiveness evaluation.

A limited number of oceanic targeted observations in real scenarios have been reported in the literature^14–16^. Curtin and Bellingham^14^ implemented the Autonomous Ocean Sampling Network (AOSN) field program in the Monterey Bay and demonstrated that proper sampling is critical for both understanding and predicting ocean fields. To predict the local ocean circulation and potential pathways of spilled oil, Shay et al.^15^ carried out oceanographic surveys based on the positions of exploded oil rig and the loop currents in the Gulf of Mexico. They found that the root-mean-square errors (RMSEs) of the simulated results were reduced by approximately 30% when the additional measurements were assimilated into the hindcast model. Guided by the optimal designed glider trajectory, which sets the trace of the error covariance matrix as criteria^17^, Mourre and Alvarez^16^ found that the data assimilation performance of the adaptive-sampling-driven glider data was better than that of the independent glider data in the same region, with a RMSEs reduction of 18%.

However, none of the abovementioned in-field oceanic targeted observations were designed based on identified “sensitive areas”. Given a certain research subject, sensitive areas are the specific localized areas that are expected to contribute most in reducing prediction uncertainties in the target region. In a study of storm tracking prediction, Montani et al.^18^ demonstrated that short-range prediction refinement can be significantly increased if the observations are deployed in sensitive areas. In the Atlantic observing-system research and predictability experiment (THORPEX), Majumdar^5^ concluded that targeting and assimilating observations in the sensitive areas are effective in improving forecasts. Targeted observation studies in the atmospheric field started earlier and are more mature than those in the ocean, field targeted observation based on sensitive areas has already been conducted^5^. To our knowledge, however, tests of targeted observations guided by identified sensitive areas in real at-sea scenarios are still lacking.

**Figure 1:**
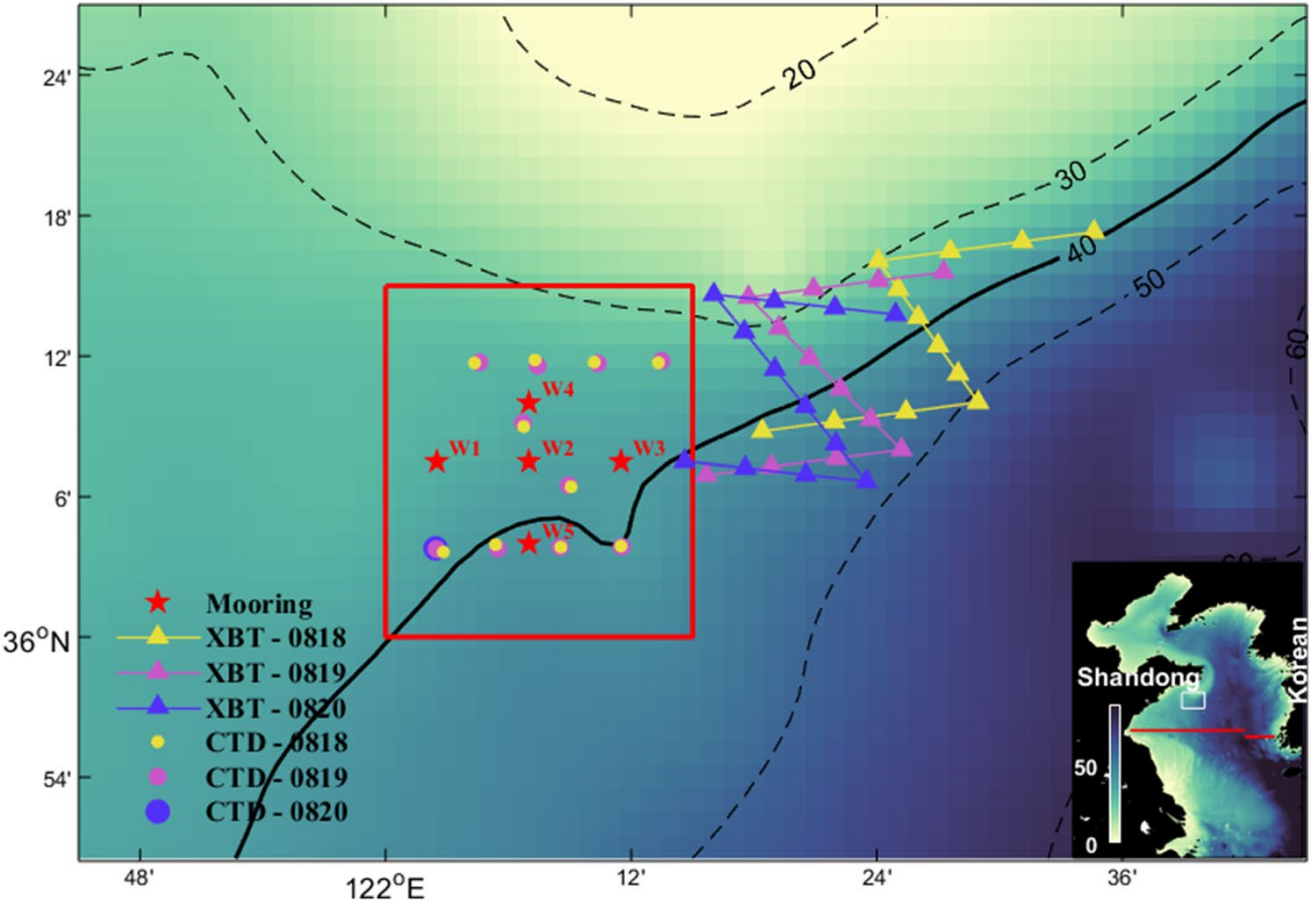
Plan view of the locations of the five temperature profile buoy stations (red stars), thirty-six XBT stations (triangles), and twenty-one shipboard CTD stations (circles). The differences in the deployment times of the XBT and shipboard CTD observations are distinguished by different colors. The red box indicates the location of the target region. The topography is indicated by shading. The bottom-right insert shows the model area, in which the write box indicates the position of the study area and the red lines indicate the section locations used for vertical thermal structure validation. Figures are plotted using MATLAB R2017a (http://www.mathworks.com/) with M_Map v1.4 (a mapping package, http://www.eos.ubc.ca/~rich/map.html).

In August 2019, an exploratory field experiment is conducted on the northwest continental slope of the Yellow Sea (YS; Fig. 1), aiming at improving the thermal structure predictions by oceanic targeted observations based on identified sensitive areas. It is expected that assimilating observations in the identified sensitive areas will be more effective than assimilating observations in other areas. Temperature is selected as the target variable because it is a key variable in controlling density fields, the vertical thermal structure can modulate the sound speed profile and has a crucial impact on the acoustic propagation^19^. The selected target region locate near the margin of the Yellow Sea Cold Water Mass (YSCWM)^20^. Under the comprehensive impact of the thermodynamic and dynamic oceanic processes and topography, the thermal structures in this region feature significant spatial and temporal variations, and their forecast uncertainty is generally large^21,22^. One main goal of this exploratory study is to simulate the future customized-rapid-prediction-supporting scenarios, thus here we focus on improving the short range (7 day) thermal structure prediction after conducting rapid targeted observation. In the present paper, we report the identification procedure of sensitive areas and design of observation strategies and validate the effectiveness of targeted observations.

The article is organized as follows: the observation data, model configuration, the Conditional Nonlinear Optimal Perturbation (CNOP) approach and the assimilation technique are briefly described in section Methods. In section Results, given a specified target region, the sensitive areas for thermal structure prediction are identified and validated. The physical mechanism behind are discussed. Then, the observation strategies are designed and quantitatively assessed by conducting a series of Observing System Simulation Experiments (OSSEs). The improvements in the thermal structure prediction due to the targeted observation through Observing System Experiments (OSEs) is also presented. The results are summarized in the last section.

## Methods

### Observation data

A dedicated ocean survey with two synergetic ships is carried out in August 2019 to obtain the targeted observation data in the YS. In the target region (red box in Fig. 1), five buoys are placed during 17–27 August for forecast validation. The buoys are composed of temperature loggers (SBE56), pressure–temperature loggers (SBE39 and RBRduo^3^) and pressure–temperature–conductivity loggers (RBRconcerto^3^), which can obtain the temperature profiles of nearly the total water volume in approximately 2 m vertical bins. Both ends of the buoys are equipped with pressure sensor instruments to determine the depths of the temperature loggers between them. The sensors collected a sample every 10 min. During 18–20 August, 21 temperature profiles are obtained in the target region by the shipboard CTD (circle stations in Fig. 1). Meanwhile, temperature profiles are collected in the identified sensitive areas by the eXpendable BathyThermographs (XBT) by the other ship. Temperature profiles at each XBT station are detected four times a day (16:30–19:30, 22:30–1:30, 4:30–7:30, 10:30–13:30) along the predesigned routes (triangle stations in Fig. 1) to obtain the daily averaged values, which are used in the cycle data assimilation on 18, 19, and 20 August 2019. All times in the study are referenced to the Chinese Standard Time (UTC + 8).

### Numerical model configuration

To investigate the utility of targeted observation in improving the prediction of thermal structures in the shallow YS, the Regional Ocean Modeling System (ROMS) was used to solve the three-dimensional Reynolds-averaged hydrostatic Navier–Stokes equation with the Boussinesq approximation^23^. The ROMS utilizes a nonlinear terrain-following vertical coordinate and has been proven to be suitable for regional ocean modeling by a large number of studies^24–27^. The K-profile parameterization scheme is used to calculate the vertical eddy viscosity and diffusivity^28^. Harmonic horizontal mixing is employed with constant horizontal eddy viscosity and diffusivity of 10 m^2^ s^−1^ and 15 m^2^ s^−1^, respectively. The bottom stress is parameterized following a quadratic formula with a constant bottom drag coefficient set to 2.5 × 10^–3^.

The model region covers the China Seas north of 30°N (Fig. 1, 30–41.3° N, 117–127° E) with 1/24° horizontal resolution, and there are 32 vertical levels that are unevenly distributed with closer spacing within the range of thermocline. The model topography is subsampled from ETOPO2 (https://ngdc.noaa.gov/mgg/global/etopo2), and the minimum water depth is set to 10 m. The model initial temperature and salinity are obtained from the multiyear averaged (1998–2018) HYCOM + NCODA reanalysis data^29^ (https://www.hycom.org/dataserver) in January. The initial current velocities and sea surface height are set to zero.

First, a climatology run is carried out from the cold start. At the open boundaries, the model is driven by the multiyear averaged monthly HYCOM + NCODA reanalysis data and tidal forcing of eight major tidal constituents (M_2_, S_2_, K_1_, O_1_, N_2_, K_2_, P_1_, and Q_1_). The tidal forcing is included at the open boundaries by the Flather condition^30^ with the tidal elevation and barotropic velocity obtained from the global inverse barotropic tidal model TPXO7.2^31^. On the surface, the wind stress, surface heat flux and water exchange are calculated from the multiyear averaged (1998–2018) monthly ECMWF ERA-Interim reanalysis data (https://apps.ecmwf.int/datasets/data/interim-full-moda/levtype=sfc/). The climatology run is integrated for 25 years for spin-up.

Thereafter, a hindcast run is conducted from January 2014 to August 2019, starting from the results of the climatology run. Twelve-hourly surface forcing from the ECMWF reanalysis data and daily boundary forcing from the HYCOM + NCODA reanalysis data are applied to drive the hindcast run. The hindcast run is also forced by tidal forcing (eight major constituents) from TPXO7.2. In this paper, the daily-averaged temperature profiles are used for analysis.

### CNOP approach for sensitive area identification

Identification of the sensitive areas is a crucial step in targeted observations^5,27^. Sensitive areas for targeted observation can be identified by the Conditional Nonlinear Optimal Perturbations (CNOP) approach proposed by Mu et al.^32^. Utilizing the CNOP approach, the optimal initial errors that cause the largest nonlinear forecast uncertainty can be calculated, and their spatial patterns help to locate the sensitive areas. To date, CNOP-identified sensitive areas have been proven to be quite effective in a number of oceanic applications, such as the prediction of the ENSO^33^, upstream Kuroshio transport^27^, Kuroshio intrusion into the SCS^24^, Kuroshio large meander^12^ and the ocean state in the SCS western boundary current region^10^.

In this section, the CNOP approach is briefly reviewed^32,34^. Let $M_{t}$ be the nonlinear propagator that propagates the value ${\mathbf{X}}_{0}$ at initial time $t_{0}$ to ${\mathbf{X}}_{t} = M_{t} ({\mathbf{X}}_{0} )$ at the end of the forecast time. When adding the initial perturbation $\Delta {\mathbf{x}}_{0}$ to the initial state, the impact of an initial perturbation $\Delta {\mathbf{x}}_{t}$ at a later time $t$ can be expressed as

1 $$ \Delta {\mathbf{x}}_{t} = M_{t} ({\mathbf{X}}_{0} + \Delta {\mathbf{x}}_{0} ) - M_{t} ({\mathbf{X}}_{0} ), $$

Following the definition proposed by Mu et al.^32^, the CNOP can be obtained by solving the following nonlinear constraint maximization problem:

2 $$ J(\Delta {\mathbf{x}}_{0,\sigma } ) = \mathop {\max }\limits_{{\left\| {\Delta x_{0} } \right\| \le \sigma }} J(\Delta {\mathbf{x}}_{0} ) = \mathop {\max }\limits_{{\left\| {\Delta x_{0} } \right\| \le \sigma }} \left\| {M_{t} ({\mathbf{X}}_{0} + \Delta {\mathbf{x}}_{0} ) - M_{t} ({\mathbf{X}}_{0} )} \right\|, $$

where $J(\Delta {\mathbf{x}}_{0} )$ is the objective function that estimates the nonlinear evolution of the perturbed model during time $t$. $\left\| . \right\|$ denotes the norm of the vector. $\Delta {\mathbf{x}}_{0,\sigma }$ is the CNOP‐type initial perturbation, which will induces the largest prediction error at the prediction time $t$. $\left\| {\Delta {\mathbf{x}}_{0} } \right\| \le \sigma$ is the constraint condition defined by the selected norm $\left\| . \right\|$.

Generally, CNOP computation relies on the adjoint technique to calculate the gradient of the objective function. However, directly calculating CNOP in a complicated model requires a considerable amount of coding and is computationally expensive^24,27,35^. Alternatively, in this study, we use an Empirical Orthogonal Function (EOF) based algorithm proposed by Wang and Tan^35^ to approximate the CNOP without using the adjoint technique (hereafter referred to as the EOF-CNOP algorithm). Wang and Tan^35^ tested the EOF-CNOP algorithm in a typhoon case, they found that the sensitive areas identified by this approximation algorithm are similar to the real CNOP results but require much less computational resources. The calculation process of the EOF-CNOP algorithm is described as follows: First, a set of initial perturbations is added to the initial state to obtain the corresponding prediction increment ensemble by numerical integration. Then, the orthogonal basis of the initial perturbation ensemble is calculated by EOF decomposition. Finally, a statistical relationship is established between the initial perturbations and the associated prediction increment; thus, the gradient of the objective function can be obtained, and the CNOP can be computed.

In practice, the specific form of the objective function and the initial constraint are defined according to the object of study. In the context of the thermal structure of interest in this study, the objective function is defined as the change in the volume-integrated temperature caused by the initial errors in the specified target region, such that

3 $$ J = \left( {\int\limits_{V} {\Delta T_{t} dxdydz} } \right)^{2} , $$

where $\Delta T_{t}$ indicates the temperature anomaly at the future time $t$ caused by the initial errors and $V$ denotes the three-dimensional water volume in the selected target region.

Following the formula of Li et al.^10^, the initial constraint is defined as

4 $$ \left\| {\Delta x_{0} } \right\|^{2} = \int\limits_{D} {(\frac{{\Delta T_{0} }}{{T_{std} }})^{2} } dxdydz \le \sigma^{2} , $$

where $\Delta T_{0}$ indicates the initial temperature perturbation, $D$ denotes the whole model domain, and $T_{std}$ indicates the regionally averaged temperature standard deviation in the model domain, which is calculated from the World Ocean Atlas 2018 (WOA18, https://www.nodc.noaa.gov/OC5/woa18/) in August and was found to be 0.25 °C in this study. After completing all these steps, the sequential quadratic programming^36^ algorithm is employed to compute the CNOP.

### Optimal interpolation data assimilation method

The Optimal Interpolation (OI) technique is used to assimilate the targeted observation data to reduce uncertainties in the initial fields, which can be formulated as

5 $$ \left\{ \begin{gathered} {\mathbf{x}}_{a} = {\mathbf{x}}_{b} + {\mathbf{K}}({\mathbf{y}}_{obs} - {\mathbf{Hx}}_{b} ) \hfill \\ {\mathbf{K}} = {\mathbf{BH}}^{T} ({\mathbf{HBH}}^{T} + {\mathbf{R}})^{ - 1} \hfill \\ \end{gathered} \right., $$

where ${\mathbf{x}}_{a}$ and ${\mathbf{x}}_{b}$ indicate the analysis field and background field, respectively. ${\mathbf{y}}_{obs}$ denotes the observation vector, and ${\mathbf{H}}$ is the observation operator, which maps from model space into observational space. ${\mathbf{K}}$ is the Kalman gain matrix, which is calculated based on ${\mathbf{H}}$, the model background field error covariation matrix ${\mathbf{B}}$, and the observational error covariation matrix ${\mathbf{R}}$. ${\mathbf{R}}$ is diagonal since all the observational errors are assumed uncorrelated in space. That is,

6 $$ {\mathbf{R}}_{ij} = \sigma_{o}^{2} \delta_{ij} , $$

where $\sigma_{o}$ is determined by the observations accuracies, $\delta_{ij}$ is the Kronecker delta, $\delta_{ij} = 1$ when $i = j$, and $\delta_{ij} = 0$ when $i \ne j$. The model background field error covariation matrix ${\mathbf{B}}$ at different vertical layers is assumed to be independent. Similar to the estimation used by Zhang et al.^13^, ${\mathbf{B}}_{ij}$ is written as follows:

7 $$ \left\{ {\begin{array}{*{20}l} {{\mathbf{B}}_{ij} = \sigma_{m}^{2} \exp ( - ({\raise0.7ex\hbox{${d_{ij} }$} \!\mathord{\left/ {\vphantom {{d_{ij} } {L_{c} }}}\right.\kern-\nulldelimiterspace} \!\lower0.7ex\hbox{${L_{c} }$}})^{2} )} \hfill & {d_{ij} \le {\mathbf{R}}_{0} } \hfill \\ 0 \hfill & {d_{ij} > {\mathbf{R}}_{0} } \hfill \\ \end{array} } \right., $$

where $\sigma_{m}$ is determined by the initial model errors, $d_{ij}$ is the distance between two model grid points $i$ and $j$. Referring to the method of Cao et al.^37^, by analyzing the distribution of the correlation coefficient with distance, the correlation length $L_{c}$ and the influence radius *R*_*o*_ were set to 60 km and 120 km, respectively. In this paper, profile data was first assimilated separately at each single depth level with constant vertical interval of 1 m and then the assimilated field is interpolated vertically to the model levels.

## Results

### Model validation

**Figure 2:**
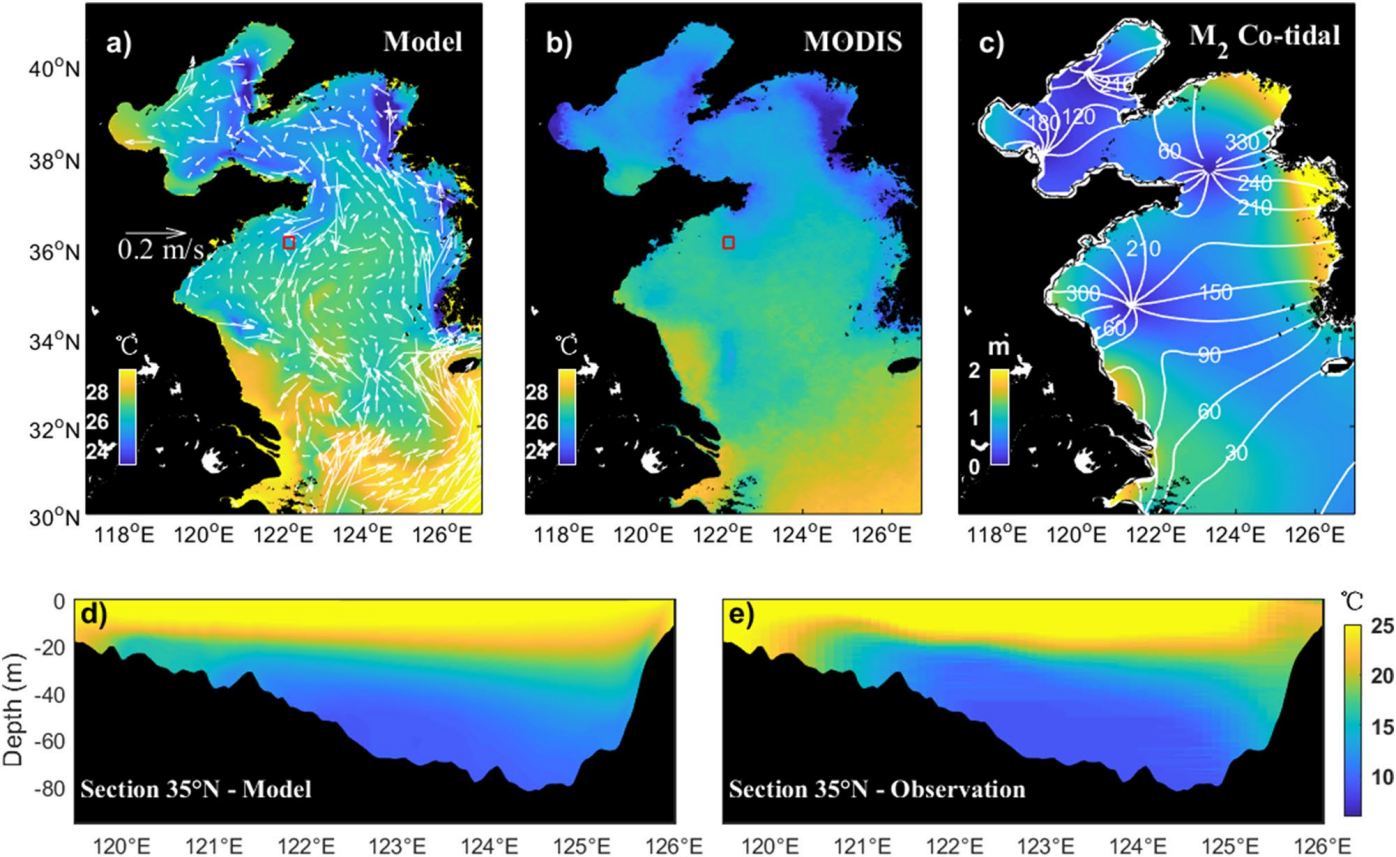
Temperature validation between the monthly-mean climatology (Aug) simulations and observations. (**a**,**b**) Comparison of the sea surface temperature between the model results and MODIS data. The red box indicates the location of the target region. The simulated current field at the depth of 10 m is also shown in (**a**). (**c**) M_2_ barotropic cotidal chart with amplitude (colors) and cophase (white lines) lines. **(d**,**e)** Comparison of the along section (35°N) temperature between the model results and the observations redrawn from the Atlas of Ocean Data in the China Seas. Figures are plotted using MATLAB R2017a (http://www.mathworks.com/) with M_Map v1.4 (a mapping package, http://www.eos.ubc.ca/~rich/map.html).

To validate the simulations, the modeled monthly averaged (August) sea surface temperature in the simulation area in the last climatology year is extracted and compared with MODIS data (Fig. 2a, b). Against the background high sea surface temperature in summer, several surface cold patches can be clearly identified along the coast of Shandong Peninsula and Korean Peninsula, indicating the occurrence of upwelling (Figs. 1, 2a). The positions of the modeled surface cold patches are generally consistent with satellite observations (Fig. 2a, b). The cotidal chart of M_2_ tide (Fig. 2c) and the anticlockwise YSCWM circulation in the middle YS are also successfully reproduced^22^. In addition, the simulated monthly averaged (August) temperature along the 35°N section (see location in Fig. 1) is also extracted and compared with previous observations obtained from the Atlas of Ocean Data in the China Seas^38^. In summer, the water is well mixed in very shallow regions near the coast and is strongly stratified in the central basin. The simulated vertical distribution of isothermals is generally consistent with observations (Fig. 2d, e). Below the thermocline, the YSCWM that formed during the previous winter can be clearly identified. In general, the simulated vertical structure shows good agreement with historical observations. However, there is still room for improvement in the accuracy of the simulated thermal structure, especially the continental slope region.

### Identification and validation of CNOP-based sensitive area

To provide guidance for the targeted observation field campaign, a vital step is the identification of the sensitive areas. In this study, identification of the sensitive areas from the real-time predicted ocean state is not attempted, as this would entail the establishment of a reliable local prediction model with forcing from a larger-scale prediction model as a prerequisite. In fact, the locations of the identified sensitive areas in this study are generally consistent in space in the hindcast and climatology years (see Supplementary Fig. S-1). The sensitive area in the last climatology year is first identified. Considering the ships' voyage schedule, the initial prediction time is set to 00:00 on 20 August (hereinafter the targeting time), and the daily averaged temperature profiles between 00:00 26 and 00:00 27 August (hereinafter the verification time) in the target region are used for the forecast validation.

Following Wang and Tan^35^, to identify the sensitive area, an ensemble of 20 initial perturbations and a nature run without perturbation is ran for the last climatology year. For this study of thermal structure prediction, initial perturbations are added to the temperature, which is achieved by taking the discrepancy of the daily averaged HYCOM + NCODA temperature on 20 August between every two adjacent years during 1998–2018. All the initial temperature perturbations are scaled so that their standard deviation is 0.25 °C. Then, the CNOP are calculated by employing a vertically integrated temperature scheme based on the total 21 sets of initial ensemble conditions and the corresponding 7th day forecast samples. We confine the CNOP-identified sensitive area as the region where the vertically integrated CNOP-type errors are larger than a certain value $\tau$. $\tau$ is determined to obtain a sensitive area of the same size as the target region.

**Figure 3:**
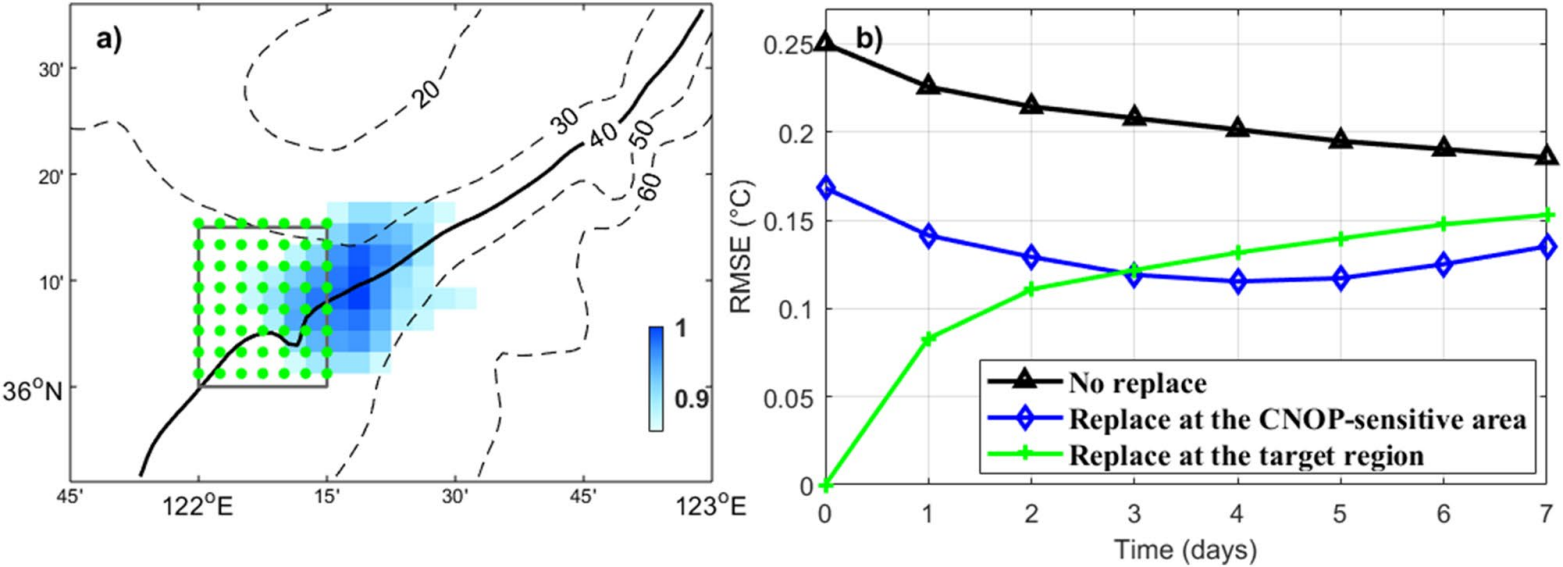
(**a**) Location of the identified sensitive area for the last climatology year (blue color). The CNOP are normalized according to their maximum value. The gray box indicates the location of the target region and the green dots denote the grid points in the target region. The 20–60 m isobaths are also shown. (**b**) Temporal evolution of the mean temperature profile RMSEs in the target region during the prediction time based on the 20 sets of replace experiments. Figures are plotted using MATLAB R2017a (http://www.mathworks.com/).

The spatial distribution of the calculated CNOP for the last climatology year is shown in Fig. 3a, the CNOP are normalized according to their maximum value. The identified sensitive area mainly locates outside of the target region in the northeast, with only a small fraction of the area overlapping. Similar results have been obtained for other climatology years (results shown in an earlier paper by Hu et al.^39^), confirming the stability of this method.

To validate the effectiveness of the CNOP-based sensitive area, a series of experiments are implemented based on the simulated results of the last climatology year. The original ocean state is denoted by the nature run EXP0, which is considered as the synthetic observation. Realistic observation errors were not additionally considered in order to isolate the impacts of synthetic observations^13^. Then, the control experiments (EXP_perturb) are created by superimposing 20 sets of random temperature perturbations with a normal distribution N(0, 0.3) °C to EXP0 at 00:00, 1 August. The perturbation magnitude is set to 0.3 °C to get an approximately 0.25 °C perturbation magnitude in target region at the targeting time. In addition to the nature run and the control run, two sets of experiments (EXP_replace_tar and EXP_replace_sen) are conducted through replacing the synthetic observations in different regions at the targeting time. It should be noted that, data assimilation technique is not used yet in these two sets of experiments. The regionally averaged temperature profile RMSEs in the target region at the verification time between the nature run EXP0 and other experiments are used to evaluate the effectiveness of the CNOP-based sensitive area.

The temporal evolutions of the mean temperature profile RMSEs based on the 20 sets of replace experiments are shown in Fig. 3b. For the control experiment (EXP_perturb), the regionally averaged RMSEs in the target region are approximately 0.25 °C at the targeting time and attenuate to approximately 0.19 °C at the verification time (the black line in Fig. 3b). In the EXP_replace_tar, which represents the conventional observation strategy, the initial RMSEs are zero in the target region (the green line in Fig. 3b). During the 7 days integration, effectiveness of the forecast refinement continuously decreases from the targeting time. In the EXP_replace_sen, the initial RMSEs are also reduced at the targeting time (the blue line in Fig. 3b) because of the overlapping between the CNOP-based sensitive area and the target region. However, at the verification time, the forecast errors in the EXP_replace_sen are smaller than that in both EXP_perturb and EXP_replace_tar. These results support the effectiveness of the CNOP-based sensitive area.

To better understand how the local forecast errors are efficiently reduced by conducting targeted observations in the remote sensitive area, it is worth exploring the underlying dynamics. The physical processes affecting the water temperature in the target region are investigated quantitively using the model temperature equation

8 $$ \frac{\partial T}{{\partial t}} = - \nabla \cdot (\vec{v}T) + \nabla_{h} (A_{h} \nabla_{h} T) + \frac{\partial }{\partial z}(A_{v} \frac{\partial T}{{\partial z}}), $$

where $T$ is temperature, $\vec{v}$ is velocity, and $A_{h}$ and $A_{v}$ are the horizontal and vertical diffusivity coefficients, respectively. The temperature change in the water is mainly induced by horizontal temperature advection, vertical temperature advection, horizontal temperature diffusion and vertical temperature diffusion. The ocean temperature is also affected by the change in surface heating. However, in this study, we only conducted targeted observations inside the water volume, thus, only the impact of advection and diffusion processes are discussed.

**Figure 4:**
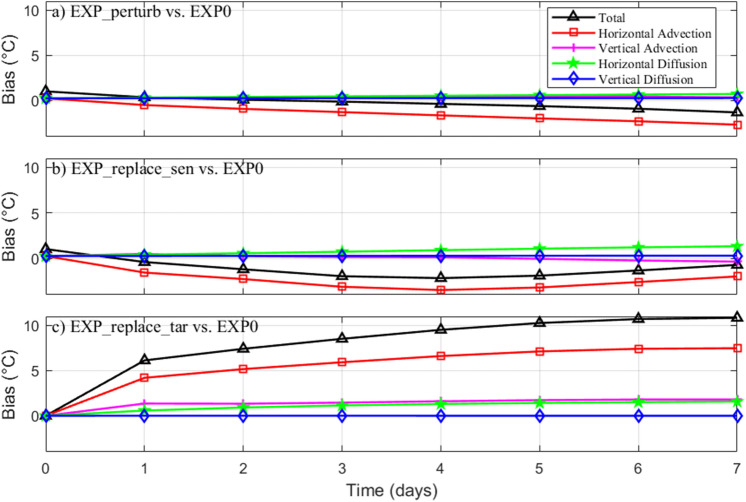
Temporal evolution of the mean vertically-integrated and regionally-averaged temperature biases during the prediction time based on the 20 sets of replace experiments induced by different processes in the target region for (**a**) EXP_perturb versus EXP0, (**b**) EXP_replace_sen vs. EXP0, and (**c**) EXP_replace_tar versus EXP0. Figures are plotted using MATLAB R2017a (http://www.mathworks.com/).

Based on the 20 sets of replace experiments, the temporal evolution of the mean vertically-integrated and regionally-averaged temperature biases in the target region induced by different processes is shown in Fig. 4. The total temperature biases magnitude for EXP_replace_tar versus EXP0 are larger than that for EXP_perturb versus EXP0 and EXP_replace_sen versus EXP0 (Fig. 4), this indicate that the temperature field change very dramatically and the forecasting effectiveness is difficult to maintain in EXP_replace_tar (Fig. 3b). It is clear that the horizontal advection accounts for the majority of the temperature biases during the prediction time. Considering that the temperature field in the targeted region are continuously improved in EXP_perturb and EXP_replace_sen (Fig. 3b), it can be inferred that the horizontal advection process makes a dominated positive contribution. In contrast, in the EXP_replace_tar, the temperature RMSEs in the target region grow continuously since the targeting time (Fig. 3b), the horizontal advection process makes the major negative effect. The contribution of vertical advection, horizontal diffusion and vertical diffusion to temperature biases is relatively small. From historical studies, in the summer YS most of the water volume is dominated by a basin-scale cyclonic gyre (approximately 0.2 Sv) as the baroclinic response of the YSCWM^22,40,41^. The identified sensitive area is located northeastward of the target region, which is consistent with the local flow direction of the YSCWM circulation (southwestward). By replacing data in the sensitive area, the information is subsequently advectively carried downstream to the target region by the YSCWM circulation.

**Figure 5:**
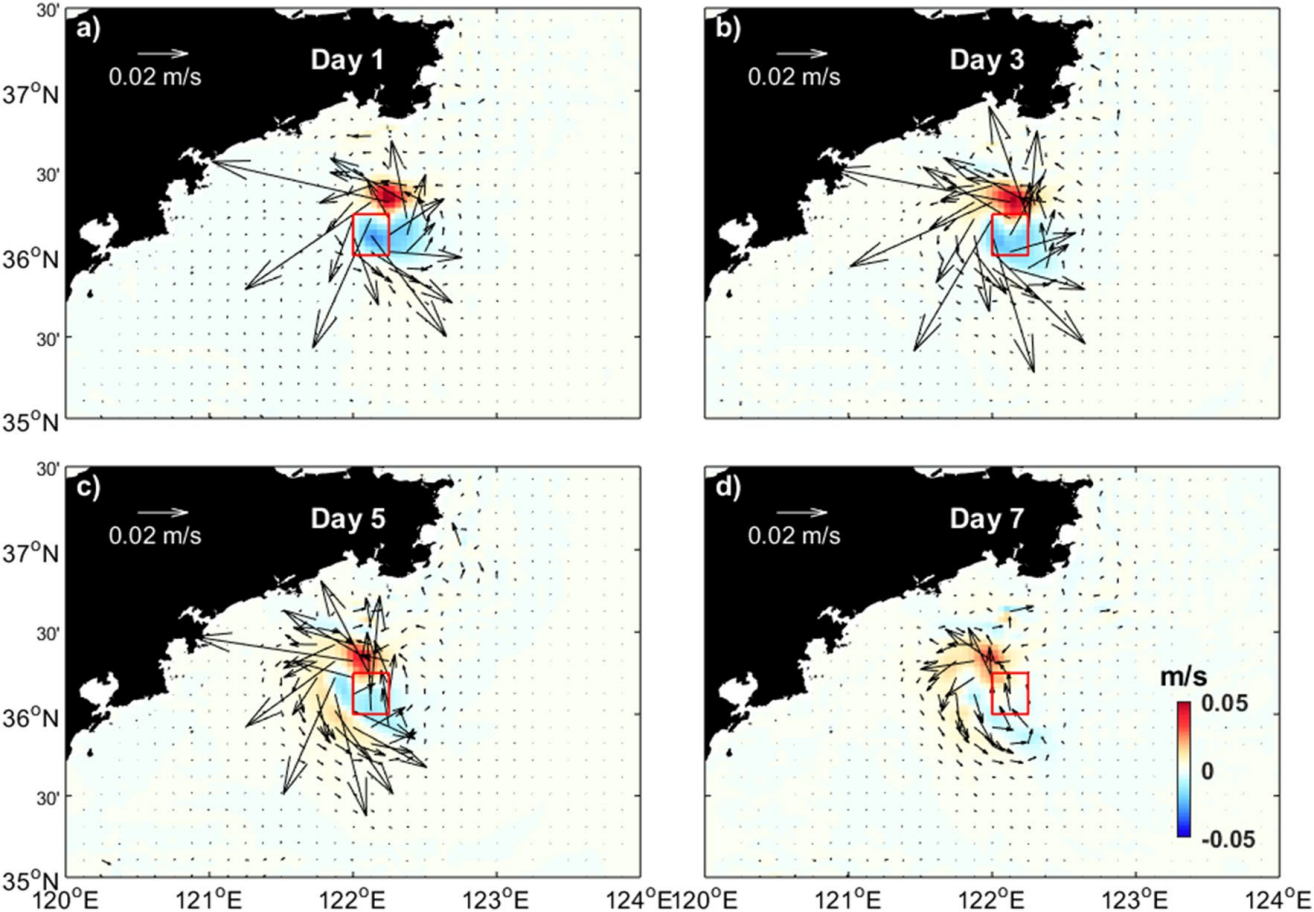
Difference in the daily averaged currents field at the depth of 10 m between an EXP_replace_sen case and EXP0 at days 1, 3, 5, and 7, respectively. The red box indicates the location of the target region. Figures are plotted using MATLAB R2017a (http://www.mathworks.com/) with M_Map v1.4 (a mapping package, http://www.eos.ubc.ca/~rich/map.html).

One strong advantage of the CNOP method is taking the nonlinearity into account in the optimization problem. Figure 5 gives the difference in the simulated current fields between an EXP_replace_sen case and EXP0 during the prediction time. By replacing temperature data in the CNOP-based sensitive area, the current fields are also changed. The temperature variation induced current change is a nonlinear process. Thus, in this study, the nonlinear horizontal temperature advection is believed to be the major mechanism dominating the temperature refinement in the target region.

### Observation strategy and benefit assessment with Observing System Simulation Experiments

Before actually starting the field campaign, a targeted observation strategy that includes the ship route and the deployment locations should be designed. Moreover, the data assimilation technique (we use OI data assimilation here) should be utilized to maximize the benefit of the limited observation resources. On the basis that the locations of the identified sensitive areas are generally consistent in space in the hindcast and climatology years (see Supplementary Fig. S-1), the CNOP-identified sensitive area from the last climatology run is used to guide the observation strategy design.

**Figure 6:**
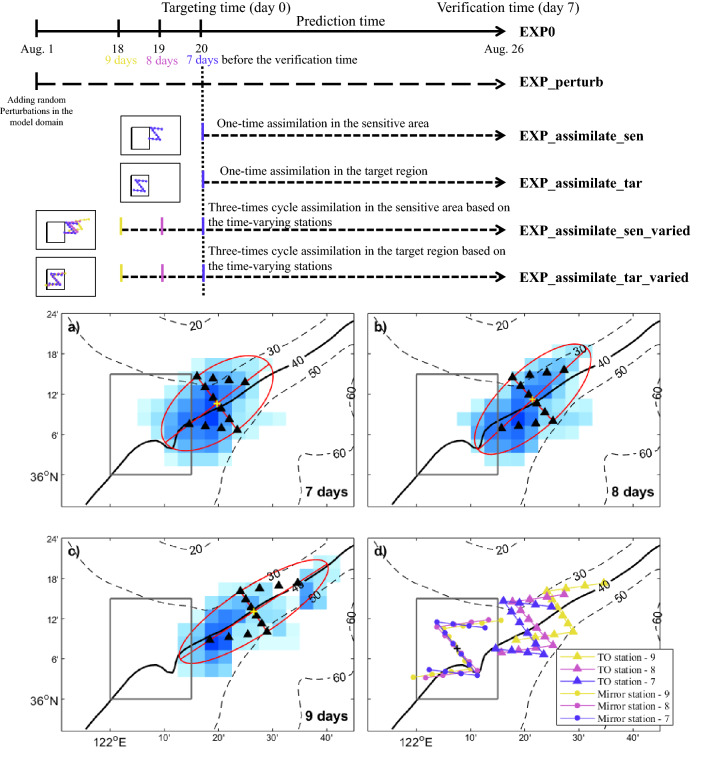
Schematic diagram of the Observing System Simulation Experiments based on the last climatology run. All the assimilation experiments use the results of the nature run as synthetic observations. The assimilated data station locations and the corresponding assimilation times are plotted by the same colors. (**a**–**c**) Z-shape observation stations (black triangles) designed based on the time-varying sensitive areas (background colors). The red ellipses are fitted to represent the most of the sensitive areas, which are used in the stations design. The gray box indicates the location of the target region. (**d**) The triangle stations denote the targeted observation (TO) stations, their locations are the same as those in (**a**–**c**), the circle stations indicate the corresponding mirror stations inside the target region. The different deployment times of the observations are distinguished by different colors. Figures are plotted using MATLAB R2017a (http://www.mathworks.com/) and Microsoft PowerPoint 2016 (https://office.live.com/start/PowerPoint.aspx).

**Figure 7:**
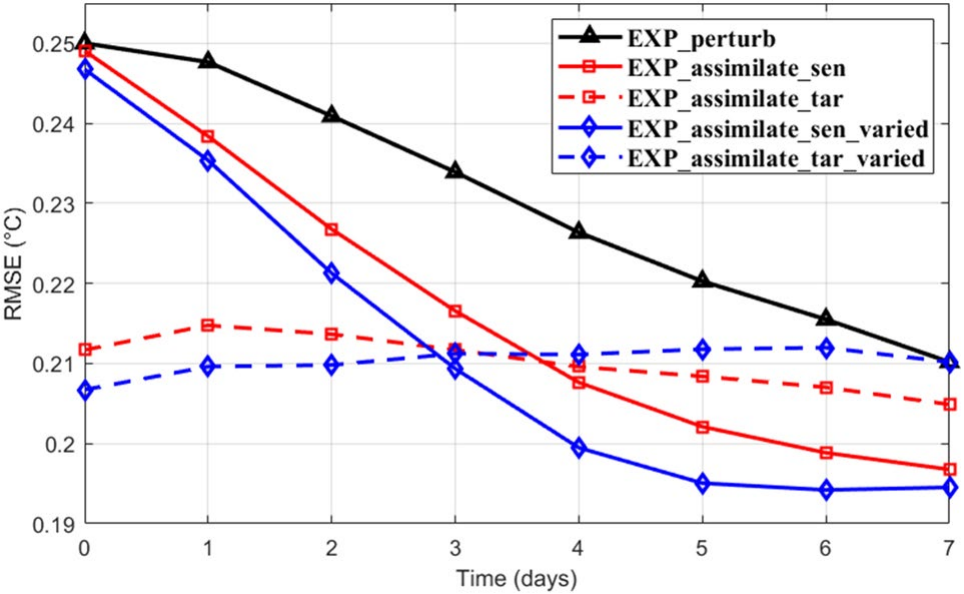
Temporal evolution of the temperature profile RMSEs in the target region during the prediction time among the Observing System Simulation Experiments. Figures are plotted using MATLAB R2017a (http://www.mathworks.com/).

To maximize the observation coverage in the sensitive area with limited observation resources, a Z-shaped observation strategy with 12 stations is designed based on the identified sensitive area (Fig. 6a, see detailed observation stations design steps in the supplemental material). It is worth noting that, this observation strategy is designed based on several subjective assumptions and may not be the best solution. Observation optimization strategies for guiding targeting observations are urgently needed but are beyond the scope of this paper and will be investigated in future studies. Except for the westernmost station, all the designed observation stations are out of the target region. To evaluate the performance of the designed observation stations and the assimilation system, a series of OSSEs are conducted based on the nature run EXP0 and the control experiment EXP_perturb with the largest prediction errors (Fig. 6). Two assimilation experiments (EXP_assimilate_sen and EXP_assimilate_tar) are conducted through assimilating the synthetic observations at the targeting time. Stations for EXP_assimilate_sen are located in the sensitive area along the designed Z-shape route (the blue triangle stations in Fig. 6d). Stations in EXP_assimilate_tar are the mirror stations of EXP_assimilate_sen, their center located in the center of the target region (the blue circle stations in Fig. 6d). The temporal evolutions of the temperature profile RMSEs during the prediction time in the OSSEs are shown in Fig. 7 (the red solid and dashed lines). Results in the assimilation experiments (EXP_assimilate_tar and EXP_assimilate_sen) are similar to that in the replace experiments (EXP_replace_tar and EXP_replace_sen), assimilating data in the sensitive area is more useful than that in the target region. This supports the effectiveness of our observation strategy and data assimilation system.

To further reduce the forecast errors, the possibilities for improving the initial state is explored by utilizing the intermittent cycling assimilation technique with 3 days observations. It is realized that the locations of the identified sensitive areas may be different with changing prediction periods. Following the same procedure, the sensitive areas which are 8 days and 9 days before the verification time are identified and shown in Fig. 6b,c. Centrals of the identified sensitive areas (yellow crosses in Fig. 6a–c, which are the mean positions of all the grid points in the sensitive areas) move northeastward and the shapes of the sensitive areas become oblate with increasing prediction time. The distance of the identified sensitive area from the target region is associated with the involved prediction time, this result is consistent with previous dynamic analysis. New deployment locations based on the identified 8-days and 9-days sensitive areas are designed following the same rule (Fig. 6b,c). All the stations based on the 8-days and 9-days sensitive areas are outside of the target region.

The impact of the cycling data assimilation based on the time-varying observation stations is evaluated by conducting two extra experiments, EXP_assimilate_sen_varied and EXP_assimilate_tar_varied. The assimilation interval and the total assimilation time are set to 1 day and 3 days, respectively. In EXP_assimilate_sen_varied, data are cycle assimilated three times (00:00, 18, 19, 20 August) at the stations of the 7-days, 8-days and 9-days sensitive areas (the triangle stations in Fig. 6d), respectively. The stations in the EXP_assimilate_tar_varied are mirror stations to that in the EXP_assimilate_sen_varied (the circle stations in Fig. 6d), with their daily averaged positions all locate in the center of the target region. At the targeting time (20 August, day 0 in Fig. 7), RMSEs in the EXP_assimilate_sen_varied (EXP_assimilate_tar_varied) are less than that of EXP_assimilate_sen (EXP_assimilate_tar), indicating the refinement of the initial field. After 7 days integration, EXP_assimilate_sen_varied performs the best among all the OSSEs in reducing the forecast error at the verification time.

To further confirm the validity of the observation strategy in the subsequent field operation, additional OSSEs based on the simulated results of the hindcast years 2016–2018 (Table 1) are also conducted. In every hindcast year, the hindcast control experiments are first created following the same procedures as those in EXP_perturb. Then, similar to EXP_assimilate_sen_varied and EXP_assimilate_tar_varied, the benefit of the targeted observation is tested through assimilating the synthetic observations at the time-varying stations in the sensitive areas and the target region, respectively. After 7 days integration, in every hindcast year, assimilating data in the sensitive areas based on the above determined observation strategy can yield more profit than the conventional local data assimilation (Table 1). All the results mentioned above support the implementation of the targeted observation campaign in the summer 2019 in the YS.

**Table 1:** Assessment of the designed observing strategy in the hindcast years of 2016–2018 (RMSEs improvement in percentage).

Experiments	Year (%)		
	2016	2017	2018
EXP_assimilate_tar_varied	− 32.0	20.3	59.7
EXP_assimilate_sen_varied	43.9	48.2	70.1

### Forecast improvements and effectiveness evaluation with Observing System Experiments

The benefit of oceanic targeted observations has been tested in some previous studies through a series of OSSEs^10,12,13^. However, the effect of oceanic targeted observations guided by the sensitive areas has never been tested in OSEs utilizing real data in actual operation. Generally, in the context of standard OSEs designed for atmospheric targeted observation, the experiment assimilating all the available observations is regarded as the control experiment, and the impact of the selected observations is assessed by removing subsets of the measurements or by adding extra measurements and comparing the results with the control experiment^42^. In the oceanic region of this study, the historical observations that we can obtained are sparse, so the non-assimilation experiment EXP2019 is set as the control experiment. The effectiveness of targeted observations is validated by comparing the forecast improvement of assimilating observations in different regions (Table 2).

**Table 2:** Design of Observing System Experiments.

Experiments	Data assimilation	Number of the assimilated data	Comment
EXP2019	No	0	Control run
EXP2019_sen	Yes	36	Cycle assimilate the XBT data at the designed stations in the sensitive area
EXP2019_tar_org	Yes	37	Cycle assimilate the original observations in the target region (15 profiles in 18 and 19 August, respectively, and 7 profiles in 20 August)
EXP2019_tar_interp	Yes	36	Cycle assimilate the interpolated data at the synthetic stations in the target region

In EXP2019_sen, observations obtained in the sensitive area are daily averaged and assimilated in the model. Given that the repeated cruises undergo inevitable spatial uncertainty, after performing data quality control, the temperature profiles obtained by both the XBT and the simultaneous buoys are interpolated to the predesigned station locations (the triangle stations in Fig. 6d). Generally, the target region is regarded as the most representative nonsensitive area, thus, in addition to EXP2019_sen, two extra experiments that assimilate approximately equal amounts of measurements inside the target region are conducted. In the EXP2019_tar_org, a total of 37 originally observed temperature profiles in the target region are assimilated (the circle, star and triangle stations inside the target region in Fig. 1). In the EXP2019_tar_interp, 36 interpolated data in a set of synthetic mirror stations in the target region are assimilated (the circle stations in Fig. 6d), the station locations are exactly the same with that in EXP_assimilate_tar_varied. The temperature profiles for data assimilation in the EXP2019_tar_interp are obtained by interpolating all the observations available on that day to the mirror stations. It should be noted that, to take full advantage of the limited observations, the shipboard CTD temperature profiles used in the OSEs are only one-time measurements instead of daily averaged values, which is a flaw of the designed OSEs.

**Figure 8:**
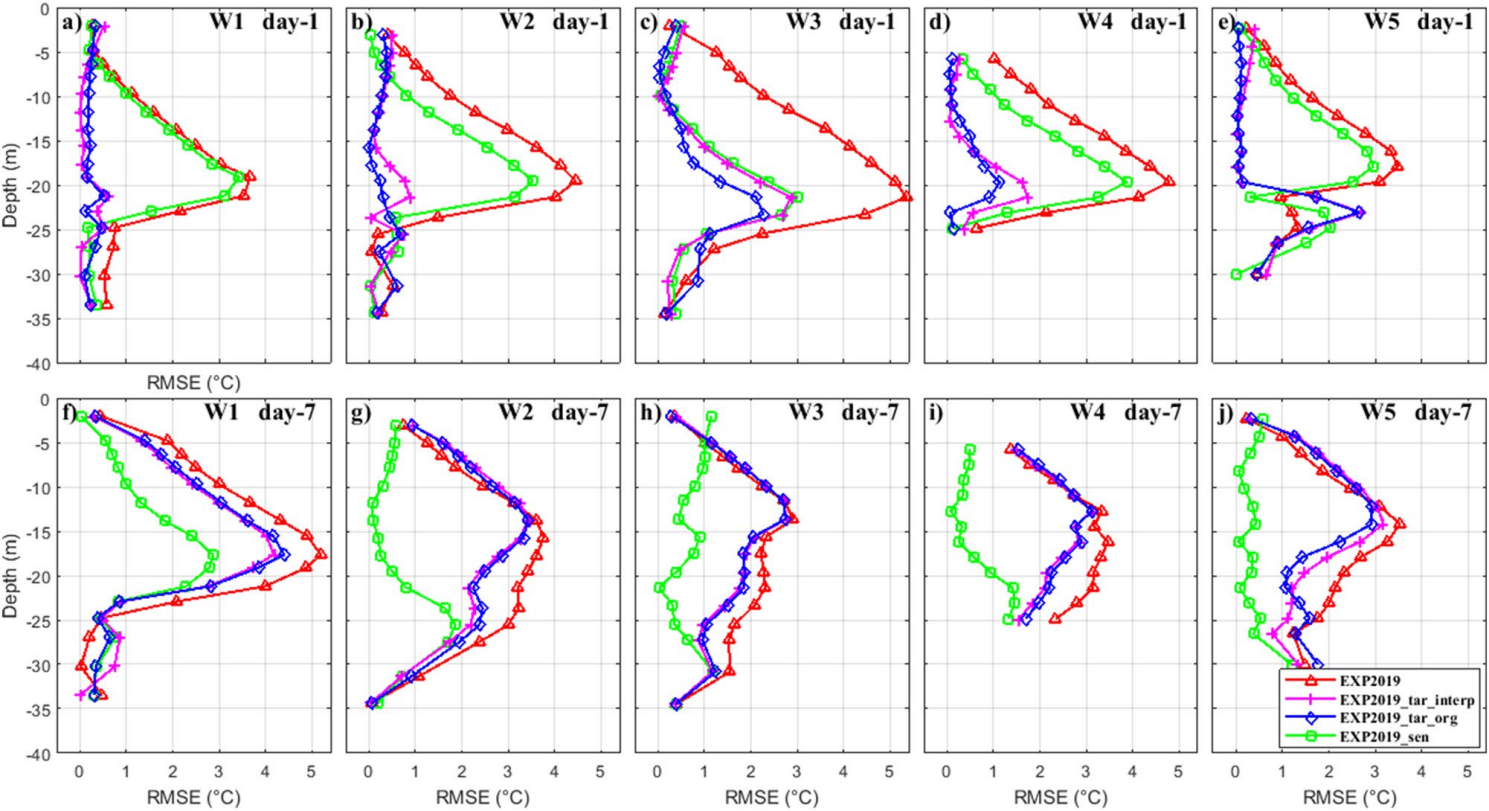
The RMSEs of daily averaged temperature profiles at five buoys between the four Observing System Experiments and the observations on the first (**a**–**e**) and 7th (**f**–**j**) day of prediction, respectively. Figures are plotted using MATLAB R2017a (http://www.mathworks.com/).

Figure 8a–e show the RMSEs of daily averaged temperature profiles at five buoys between the OSEs and the observations on the first forecast day. The temperature RMSEs are only calculated at depths where observations are available. Without data assimilation, the RMSEs between the modeled temperature profiles and the observations are approximately 1.93–3.09 °C (an average value of 2.46 °C), indicating that the simulation generally reproduced the main vertical thermal structures in the target region. In EXP2019_tar_org and EXP2019_tar_interp, the forecast improvements are nearly the same despite the difference in the spatial locations and numbers of the temperature profiles used in the cycle data assimilation (Table 2), the RMSEs are greatly reduced to approximately 0.27–1.27 °C (an average value of 0.69 °C) by assimilating local data. In contrast, the RMSEs are only slightly reduced in EXP2019_sen, because most of the assimilated data stations are out of the target region. There is one exception in station W3, where the RMSEs are all greatly reduced among the three assimilation experiments. One possible reason is that station W3 is very close to the identified sensitive area.

**Figure 9:**
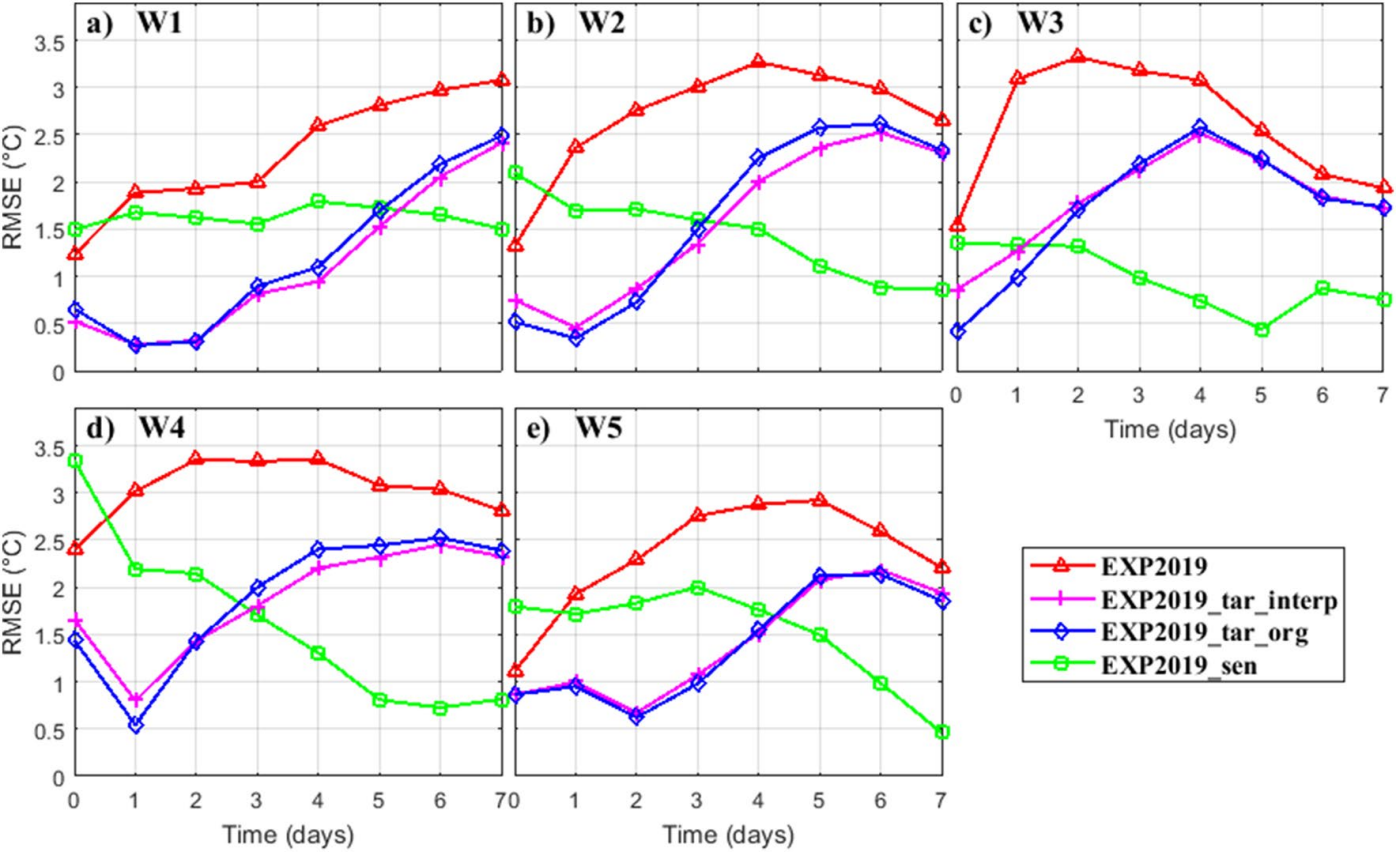
Temporal evolution of the vertically-averaged temperature profile RMSEs at five buoys between the four Observing System Experiments and the observations. Figures are plotted using MATLAB R2017a (http://www.mathworks.com/).

After 7 days integration since conducting data assimilation, the forecast improvement change remarkably among the OSEs (Fig. 8f–j). The benefit of local data assimilation (EXP2019_tar_org and EXP2019_tar_interp) remains but becomes very weak. However, in EXP2019_sen, there is a marked improvement in the vertical thermal structure predictions at the verification time (average RMSEs decrease from 2.02 to 0.88 °C, compared to the EXP2019). Figure 9 gives the temporal evolution of the vertically-averaged temperature profile RMSEs during the prediction period. Among the five buoy stations, the forecasting improvement is generally continuously decrease after conventional local data assimilation (EXP2019_tar_org and EXP2019_tar_interp). Assimilating data in the identified sensitive areas perform mediocre at the initial time, however, it yield more profit at the verification time. The results of these OSEs support our initial assumption that conducting data assimilation in the CNOP-identified sensitive area is more effective in forecast improvement at the verification time than in other areas including the target region itself. It should be noted that, the quantitative benefit of targeted observation in the CNOP-identified sensitive area could differ from model to model and may also depend on the initial simulation accuracy and the selected data assimilation scheme.

## Summary

Targeted observation is believed to be a cost-effective way to decrease forecast uncertainty through the assimilation of additional measurements into the initial state. This study first extends the scope of oceanic targeted observations to the vertical thermal structure predictions, and validate the effectiveness of targeted observation utilizing real data in actual operation. Given a selected target region and a fixed prediction period of 7 days, the sensitive areas are identified utilizing the CNOP method and a newly defined objective function. The majority of the sensitive areas are located outside of the target region in the northeast. Through conducting a series of experiments, the initial state of the CNOP-based sensitive area is proven to have the most impact on the 7th day thermal structure prediction in the target region. A term-by-term analysis of the model temperature equation indicates that, after conducting targeted observation in the upstream sensitive area, the physical signals are subsequently carried downstream to the target region by the nonlinear horizontal temperature advection of the YSCWM circulation.

Guided by the CNOP-identified sensitive area, an observation strategy is designed with the technique of cycle data assimilation and the new concept of the time-varying sensitive area. A series of OSSEs are conducted to assess the observation performance. A choreographed field campaign is then applied in the summer of 2019 in the YS to evaluate the capabilities of targeted observations. The results of OSEs show that reducing the initial errors in the sensitive area can lead to a greater improvement at the verification time than that in the target region.

In this study, we skip the step of establishing a real-time prediction model, on the basis that the locations of the identified sensitive areas in the hindcast and climatology runs are generally consistent. Although this kind of spatial consistency was also found in the optimal precursor study of the Kuroshio intrusion into the SCS (Liang et al.^24^; personal communication), it will not always be applicable if the focused phenomenon or study area changes. Thus, future work should be guided based on a reliable local prediction system. Furthermore, the optimal deployment network could be investigated and the sensitive area identification could be extended to three-dimensions. A more advanced data assimilation technique is also preferred to better exploit the targeted data.

## Supplementary Information

Supplementary Information.
