# Supplementary material for: Improving the thermal structure predictions in the Yellow Sea by conducting targeted observations in the CNOP-identified sensitive areas
Source: Sci Rep. 2021 Sep 30;11:19518. doi: 10.1038/s41598-021-98994-7 (PMC8484458; doi:10.1038/s41598-021-98994-7)
Supplement: Supplementary file 1 — Supplementary Information. [file 41598_2021_98994_MOESM1_ESM.pdf]

Improving the Thermal Structure Predictions in the Yellow Sea by Conducting Targeted Observations in the CNOP-identified Sensitive Areas

Kun Liu[1,+], Wuhong Guo[1,2,+], Lianglong Da[1,2,*], Jingyi Liu[1,2], Huiqin Hu[1], Baolong Cui[1,2]

[1]Qingdao National Laboratory for Marine Science and Technology, Qingdao, China

[2]Navy Submarine Academy, Qingdao, China

*corresponding.author Lianglong Da (da_lianglong@126.com)

[+]these authors contributed equally to this work

Comparisons of the identified sensitive area in the hindcast and climatology years

To further provide guidance for the field campaign in August 2019, the sensitive areas in the August of the last three hindcast years (2016-2018) are identified and shown in Fig. S-1. The absolute values of the CNOPs are different in every hindcast year, but only the relative values matter in the sensitive area identification; thus, the CNOPs are all normalized according to their maximum values. The locations of the maximum values are generally consistent in the hindcast and climatology years. The discrepancies among the identified sensitive areas are mainly concentrated in the marginal areas.

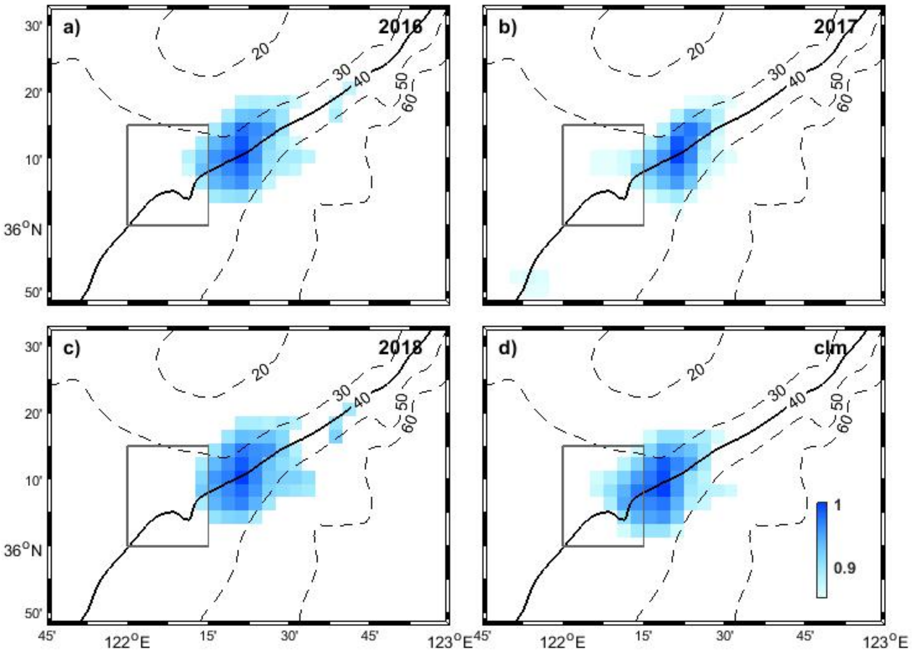

Figure S-1. Locations of the identified sensitive areas for a) b) c) the hindcast years 2016-2018 and d) the last climatology year. The CNOPs are all normalized according to their maximum values. The gray box indicates the location of the target region. Figures are plotted using MATLAB R2017a (http://www.mathworks.com/).

Design of the observation stations

Three preconditions are assumed before determining the observation stations. First, a Z-shape route is chosen to maximize the observation coverage in the identified sensitive area after conducting several numerical experiments. Second, the daily averaged temperature observations are used for data assimilation to better represent the general vertical thermal structure. To obtain the daily average temperature profiles at each station, the ship route is designed to repeat four times a day (04:30-07:30, 10:30-13:30, 16:30-19:30, and 22:30-01:30). Considering the observation simultaneity, the ship route length L is limited by the ship's speed (set to 8 knots) and the sailing time to complete each path (set to 3 hours). Third, although the prediction errors are expected to decrease for a higher number of observations, 12 stations are set along each route (approximately 4 km between the adjacent two stations) considering both the horizontal resolution (approximately 5 km) of our model and the observation cost.

Based on the above preconditions, the specific ship route and the corresponding deployment locations along it are designed as follows: First, the spatial central point of the route is determined by averaging all the model grid coordinates in the sensitive area (the yellow cross in Fig. S-2). Then, an ellipse is fitted with the central point, a major axis A_{long}, a minor axis A_{short} and a dip angle, and is scaled by a certain ratio to represent most of the sensitive area (the red ellipse in Fig. S-2). Next, six equally spaced stations are set along the minor axis (green circles in Fig. S-2). We assume that the shape "Z" is symmetric and that both ends of "Z" are located on the major axis of the ellipse. Given L and A_{short}, the leading and trailing observation stations on the major axis can be confirmed based on the Pythagorean theorem (yellow circles in Fig. S-2). Finally, four equally spaced stations are added along the other two sides of the "Z" based on the above determined stations (Fig. S-2). Except for the westernmost station, all the designed observation stations based on the identified sensitive areas 7 days before the verification time are out of the range of the target

region. Similarly, the observation stations based on the identified 8-days and 9-days sensitive areas are designed following the same rule (Fig. S-2 bc).

It is worth noting that, the settings mentioned above represent a somewhat subjective strategy based on several assumptions and may not be the best solution. Observation optimization strategies for guiding targeting observations are urgently needed but are beyond the scope of this paper and will be investigated in future studies.

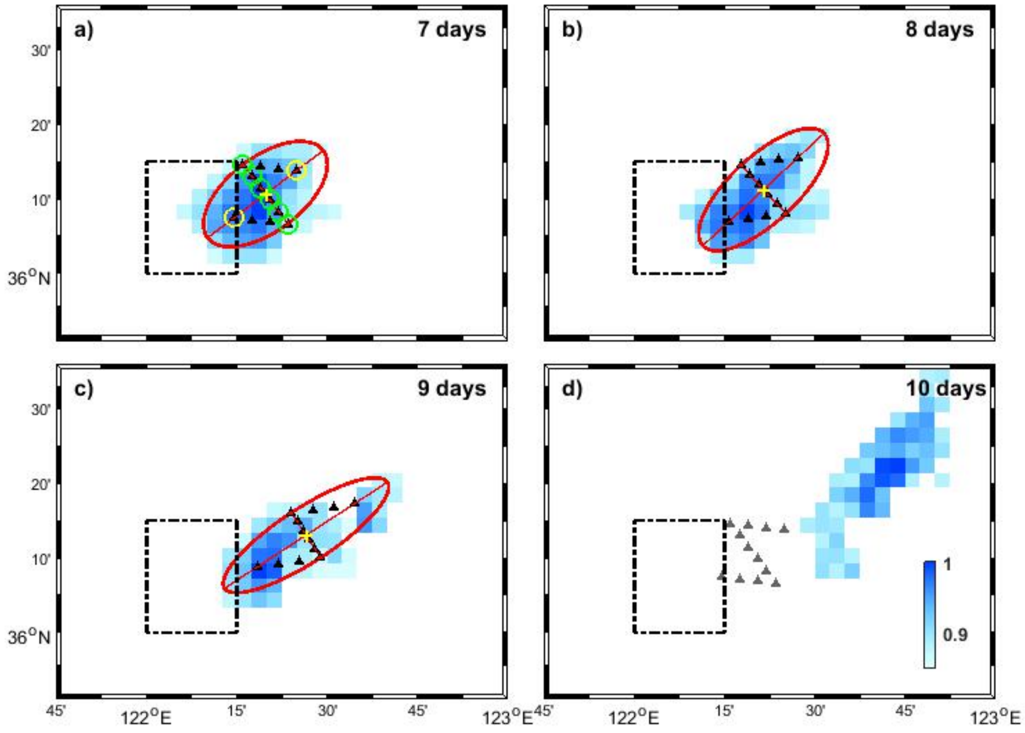

Figure S-2. a) b) c) Z- shaped observation stations (black triangles) designed based on the time-varying sensitive area (background colors). The black dashed box indicates the target region. The station locations in d) (gray triangles) are the same as those in a), which are completely out of the range of the 10-day sensitive area. Figures are plotted using MATLAB R2017a (http://www.mathworks.com/).
